# Neutrophil-to-Lymphocyte Ratio and Platelet-to-Lymphocyte Ratio as Predictors of Complicated Acute Appendicitis in Children: A Retrospective Cohort Study

**DOI:** 10.7759/cureus.115605

**Published:** 2026-09-01

**Authors:** Abdullah M Zahi, Adel A Alfayez, Bilal Qayyum, Jamal Al Hudhaif, Abdullah M Alotaibi, Mohammed T Almohaidly, Mohammad S Alonazi, Mohamad H Elmahmoud, Abdullah Abduldaem

**Affiliations:** 1 Department of Pediatric Surgery, Prince Sultan Military Medical City, Riyadh, SAU

**Keywords:** acute appendicitis, appendectomy, children, diagnostic value, neutrophil-to-lymphocyte ratio, platelet-to-lymphocyte ratio

## Abstract

Introduction: Acute appendicitis is a common pediatric emergency, but its severity is difficult to establish since conventional clinical, laboratory, and imaging methods are often imprecise, leading to unnecessary investigations or delayed care. Recent results suggest that basic inflammatory markers, especially neutrophil-to-lymphocyte ratio and platelet-to-lymphocyte ratio, may be beneficial in predicting severe appendicitis, although pediatric-specific data are still scarce. This study seeks to investigate the predictive value of these ratios for complications in children, compare their diagnostic accuracy, and determine if the inclusion of these markers in routine assessment can reduce negative appendectomy rates and enhance clinical decision-making.

Methods: The study was conducted at Prince Sultan Military Medical City from September 2021 to January 2024. All pediatric patients with a diagnosis of acute appendicitis were included. Descriptive analysis was primarily done. Categorical variables were presented as frequencies and percentages, and the chi-squared test was used to compare both groups, with p-values, degrees of freedom, and odds ratios reported. The analysis was preceded by an ethical permission from the institutional review board of the hospital.

Results: We included a total of 211 children who had undergone laparoscopic appendectomy. Of these, 176 (83.4%) had histologically confirmed appendicitis, and 35 (16.6%) had negative appendectomies. The neutrophil-to-lymphocyte ratio was significantly greater in the confirmed appendicitis patients, but the platelet-to-lymphocyte ratio did not indicate any significant difference. Both ratios were significantly higher in complicated acute appendicitis among confirmed cases. The receiver operating characteristic curve study revealed that neutrophil-to-lymphocyte ratio had higher discriminatory accuracy than platelet-to-lymphocyte ratio (area under the curve (AUC): 0.714 vs. 0.690), with both having intermediate sensitivity and specificity. These data suggest that ratios are a useful supplementary biomarker for the prediction of complex appendicitis in children.

Conclusion: Neutrophil-to-lymphocyte ratio and platelet-to-lymphocyte ratio can be beneficial in the diagnosis of complicated acute appendicitis, with neutrophil-to-lymphocyte ratio being more accurate. Nevertheless, both should be used together with clinical and radiographic assessment.

## Introduction

Acute appendicitis is one of the most common causes of abdominal pain leading to emergency surgical intervention in the pediatric population. Diagnosis is typically a considerable clinical challenge due to overlapping symptoms and variable presentation. Prompt surgical therapy is important to minimize complications such as perforation, abscess formation, and peritonitis. However, it is still important for physicians to distinguish between complicated and non-complicated cases before the operation.

Current diagnostic modalities, including clinical assessment, imaging, and laboratory testing, have insufficient specificity and sensitivity that may lead to negative appendectomies or delays of care in some circumstances. Recently, attention has been concentrated on finding accurate and accessible biomarkers that could assist in predicting the severity of the disease and guide management decisions [1]. These include the neutrophil-to-lymphocyte ratio (NLR) and platelet-to-lymphocyte ratio (PLR) which have been identified as potential inflammatory markers for being cost-effective, easy to measure, and associated with systemic inflammatory response documented in infectious and inflammatory diseases. According to several worldwide studies, high levels of NLR and PLR are associated with a greater severity of appendiceal inflammation and could be helpful in predicting complicated appendicitis, especially in children when clinical evaluation may be more difficult [2].

However, data on pediatric populations are still scarce, and further studies are needed to establish the diagnostic and prognostic value of these biomarkers in different clinical settings.

The present retrospective cohort study was designed to evaluate the predictive value of NLR and PLR in children with acute appendicitis, to assess their association with disease complications, to compare their diagnostic accuracy, and to examine their potential role in reducing negative appendectomies. Moreover, it will add to the valuable local and international data, improve comprehension of the clinical significance of these biomarkers, and investigate their incorporation into standard diagnostic protocols for better outcomes in the management of pediatric appendicitis.

## Materials and methods

Study design

This study was conducted at Prince Sultan Military Medical City (PSMMC) in Riyadh, Saudi Arabia, from September 2021 to January 2024. All children aged up to 14 years diagnosed with appendicitis who were treated at PSMMC were included in the study. The chi-squared test and odds ratio (OR) rates were used for assessments. Data were analyzed using IBM SPSS Statistics for Windows, Version 26.0 (IBM Corp., Armonk, New York, United States), with a confidence interval of 95%, processed after getting ethical approval from the Institutional Review Board of Prince Sultan Military Medical City Scientific Research Center (approval number: E-2404).

Data collection

Data of interest were retrieved from the medical files of our study subjects and entered into Excel sheets, including medical history, age, and gender, clinical data (abdominal pain, nausea/vomiting, anorexia, fever, change in bowel movement, dysuria, and recent history of upper respiratory tract infection (URTI)), and laboratory data (white blood cell (WBC) count, platelet count, lymphocyte count, and absolute neutrophil count).

Definitions

The following terms were defined as follows: non-complicated acute appendicitis, evident on histopathological examination, with features of inflammation alone; complicated acute appendicitis, evident on histopathological examination, with necrotic segments in the completely gangrenous or perforated appendix; and negative appendectomy, evident on histopathological examination as a normal appendix.

Laboratory findings

The NLR and PLR were measured in this study, and the higher values reflect an acute infection or a complicated acute appendicitis. All laboratory investigations were performed when patients presented to the emergency department before medical management was initiated.

Statistical analysis

Microsoft Excel 2010® (Microsoft Corporation, Redmond, Washington, United States) was used for data collection, storage, and management, while data coding and analysis and preparation of figures were done using IBM SPSS Statistics for Windows, Version 26.0. Descriptive analysis was primarily done. Categorical variables were presented as frequencies and percentages, and the chi-squared test was used to compare both groups, with p-values, degrees of freedom, and odds ratios reported. For continuous variables, tests for normality were done using the Shapiro-Wilk test and Q-Q plots. Normally distributed data were reported as mean and standard deviation (SD) and range, and the independent t-test was used for comparison between groups, while non-normally distributed data were presented as median (interquartile range (IQR)), and the Mann-Whitney U test was used for comparison. Any output with a p-value below 0.05 was interpreted as an indicator of statistical significance.

## Results

A total of 211 children who underwent laparoscopic appendectomy for suspected acute appendicitis were included. Patients were classified according to the final histopathological diagnosis into those with confirmed acute appendicitis (complicated and non-complicated; n=176 (83.4%)) and those with negative appendectomies (n=35 (16.6%)). The median NLR was significantly higher in patients with confirmed acute appendicitis than in those with negative appendectomy (6.6 vs. 3.7; p=0.024). In contrast, the median PLR was comparable between the two groups (188.9 vs. 183.3; p=0.685) (Tables 1-2).

**Table 1:** Demographics and baseline clinical characteristics: complicated and non-complicated appendicitis vs. negative appendectomy (no appendicitis) (n=211) *Statistically significant at a 5% level of significance; df=1 for all chi-squared tests.

Characteristic	Whole study group (n=211)	Group 1 complicated and non-complicated appendicitis (n=176; 83.4%)	Group 2 negative appendectomy (no appendicitis) (n=35; 16.6%)	P-value	Odds ratio (95% CI)
Age in years, median (IQR)	10 (8-12)	10 (8-12)	10 (7-11)	0.304	-
Gender, n (%)					
Male	124 (58.8)	106 (60.2)	18 (51.4)	0.334	1.43 (0.69-2.96)
Female	87 (41.2)	70 (39.8)	17 (48.6)		
Length of stay in days, median (IQR)	1 (1-2)	2 (1-3)	1 (1-1.3)	0.002*	-
Clinical findings, n (%)					
Abdominal pain	211 (100)	176 (100)	35 (100)	-	-
Nausea/vomiting	185 (87.7)	159 (90.3)	26 (74.3)	0.008*	3.24 (1.31-8.03)
Anorexia	170 (81.0)	143 (81.7)	27 (77.1)	0.530	1.32 (0.55-3.18)
Fever	95 (45.0)	82 (46.6)	13 (37.1)	0.305	1.48 (0.70-3.12)
Constipation	25 (11.8)	19 (10.8)	6 (17.1)	0.289	0.39 (0.22-1.59)
Diarrhea	41 (19.4)	34 (19.3)	7 (20.0)	0.926	0.96 (0.39-2.38)
Dysuria	9 (4.3)	7 (4.0)	2 (5.7)	0.646	0.68 (0.14-3.44)
History of recent upper respiratory tract infection	24 (11.4)	18 (10.3)	6 (17.1)	0.244	0.55 (0.20-1.51)
Baseline laboratory findings					
White blood cell pre-op, median (IQR)	14 (9.8-18.2)	14.6 (10.8-18.6)	9.2 (7.7-14.3)	<0.001*	-
Lymphocytes, median (IQR)	1.6 (1.1-2.4)	1.6 (1.1-2.4)	1.8 (1.1-2.7)	0.808	-
Neutrophils, median (IQR)	10.7 (6.8-14.9)	11.6 (7.6-15.4)	6.6 (4.1-11.3)	<0.001*	-
Neutrophil-to-lymphocyte ratio, median (IQR)	6.4 (3.3-11.9)	6.6 (3.7-12.3)	3.7 (2.1-9.7)	0.024*	-
Platelets, median (IQR)	323 (276-370)	322.5 (276-376.5)	326 (275-366)	0.803	-
Platelet-to-lymphocyte ratio, median (IQR)	188.5 (135-277.1)	188.9 (139.4-282.1)	183.3 (115.4-263.3)	0.685	-

**Table 2:** Demographics and baseline clinical characteristics: complicated vs. non-complicated appendicitis (n=176) *Statistically significant at a 5% level of significance; df=1 for all chi-Squared tests.

Characteristic	Group 1 complicated appendicitis (n=40)	Group 2 non-complicated appendicitis (n=136)	P-value	Odds ratio (95% CI)
Age in years, median (IQR)	11 (8-12)	10 (8-12)	0.836	-
Gender, n (%)				
Male	23 (57.5)	83 (61.0)	0.688	0.86 (0.42-1.77)
Female	17 (42.5)	53 (39.0)		
Length of stay in days, median (IQR)	5 (4-6)	1 (1-2)	<0.001*	-
Clinical findings, n (%)				
Abdominal pain	40 (100)	136 (100)	-	-
Nausea/vomiting	39 (97.5)	120 (88.2)	0.125	5.20 (0.67-40.49)
Anorexia	38 (95.0)	105 (77.8)	0.011*	5.43 (1.24-23.82)
Fever	26 (65.0)	56 (41.2)	0.008*	2.65 (1.27-5.53)
Constipation	7 (17.5)	12 (8.8)	0.120	2.19 (0.80-6.01)
Diarrhea	10 (25.0)	24 (17.6)	0.300	1.56 (0.67-3.61)
Dysuria	2 (5.0)	5 (3.7)	0.658	1.38 (0.26-7.39)
History of recent upper respiratory tract infection	5 (12.5)	13 (9.6)	0.600	1.34 (0.45-4.02)
Baseline laboratory findings				
White blood cell pre-op, median (IQR)	17.7 (13.8-20.7)	14 (10.5-17.9)	0.001*	-
Lymphocytes, median (IQR)	1.2 (1.1-1.8)	1.8 (1.1-2.5)	0.004*	-
Neutrophils, median (IQR)	14.4 (11-18)	10.5 (7.2-14.3)	0.001*	-
Neutrophil-to-lymphocyte ratio, median (IQR)	12.8 (6.5-17.2)	5.8 (3.3-9.6)	<0.001*	-
Platelets, median (IQR)	336.5 (285.3-402.8)	318 (270.3-373.3)	0.146	-
Platelet-to-lymphocyte ratio, median (IQR)	256.4 (181.8-351.5)	172.3 (131.2-251.8)	<0.001*	-

Patients with negative appendectomies were excluded, and only children with histologically confirmed appendicitis were analyzed. Patients with complicated appendicitis were compared with those with non-complicated appendicitis. Both NLR and PLR were significantly higher in the complicated appendicitis group. The median NLR was 12.8 in the complicated group compared with 5.8 in the non-complicated group (p<0.001). Likewise, the median PLR was significantly higher in patients with complicated appendicitis than in those with non-complicated appendicitis (256.4 vs. 172.3; p<0.001) (Tables 3-4 and Figures 1-2).

**Table 3:** Receiver operating characteristic curve analysis for lab results differentiating complicated and non-complicated appendicitis vs. negative appendectomy (no appendicitis)

Predictors	Area under the curve	P-value	Cut-off	Sensitivity (95% CI)	Specificity (95% CI)	LR+	LR-
Leukocyte count	0.719	<0.001	13.46	60.2 (51-69)	71.4 (63-80)	0.78	0.56
Neutrophil count	0.701	<0.001	9.99	62.5 (54-71)	68.6 (60-78)	1.81	0.55
Neutrophil-to-lymphocyte ratio	0.621	0.025	6.27	54.0 (45-63)	62.9 (54-72)	1.01	0.73
Platelet-to-lymphocyte ratio	0.522	0.685	165.48	58.5 (50-67)	48.6 (40-57)	1.16	0.85

**Table 4:** Receiver operating characteristic curve analysis for lab results differentiating complicated vs. non-complicated appendicitis

Predictors	Area under the curve	P-value	Cut-off	Sensitivity (95% CI)	Specificity (95% CI)	LR+	LR-
Leukocyte count	0.669	0.001	16.03	60.0 (51-69)	64.7 (56-74)	0.93	0.62
Neutrophil count	0.678	0.001	13.09	65.0 (56-74)	70.6 (62-79)	0.92	0.50
Neutrophil-to-lymphocyte ratio	0.714	<0.001	9.85	62.5 (54-71)	76.5 (68-85)	0.82	0.49
(Platelet-to-lymphocyte ratio	0.690	<0.001	216.22	60.0 (51-69)	68.4 (60-77)	0.88	0.58

**Figure 1:**
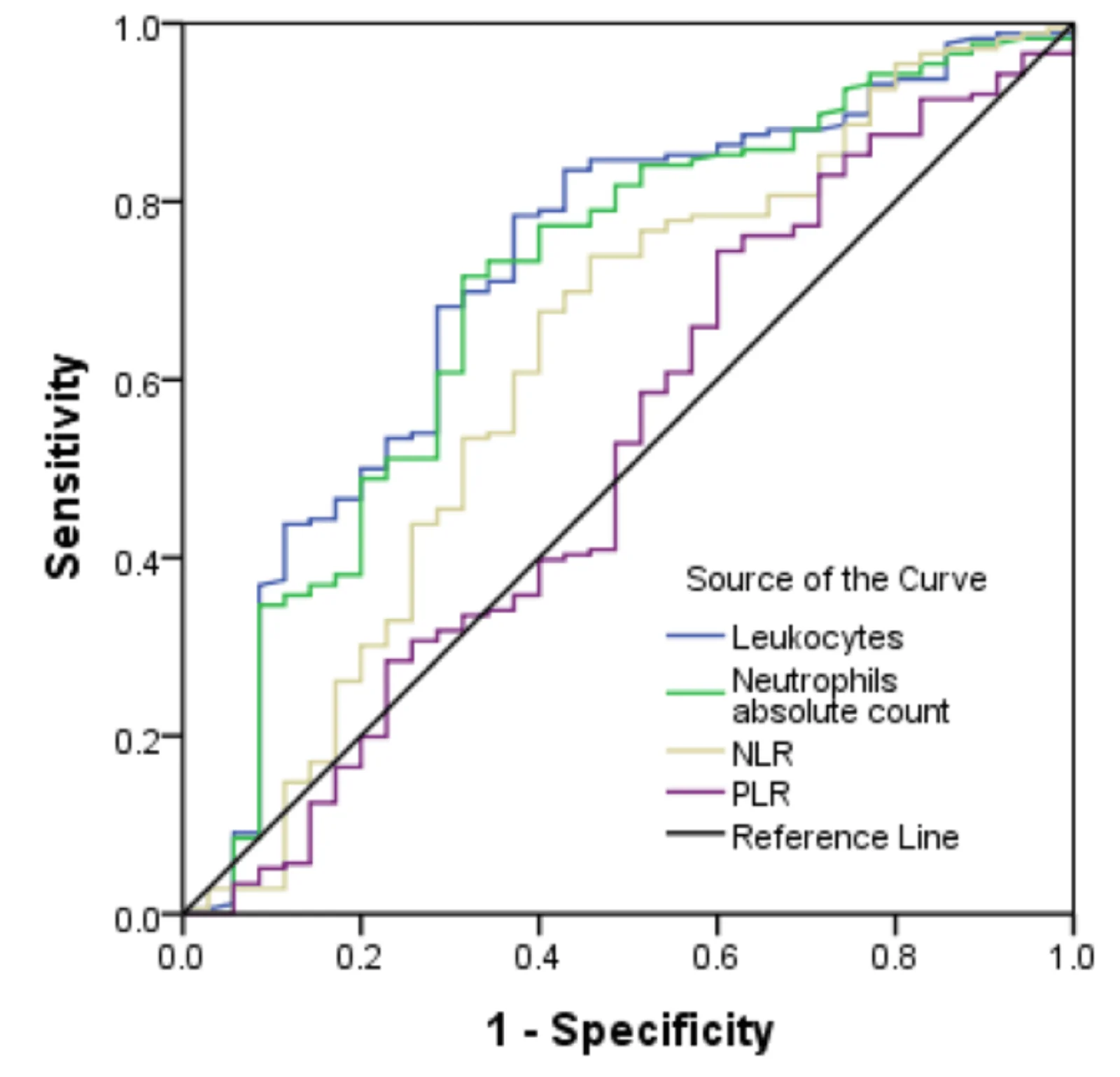
Receiver operating characteristic curve analysis for lab results differentiating complicated and non-complicated appendicitis vs. negative appendectomy (no appendicitis) NLR: neutrophil-to-lymphocyte ratio; PLR: platelet-to-lymphocyte ratio

**Figure 2:**
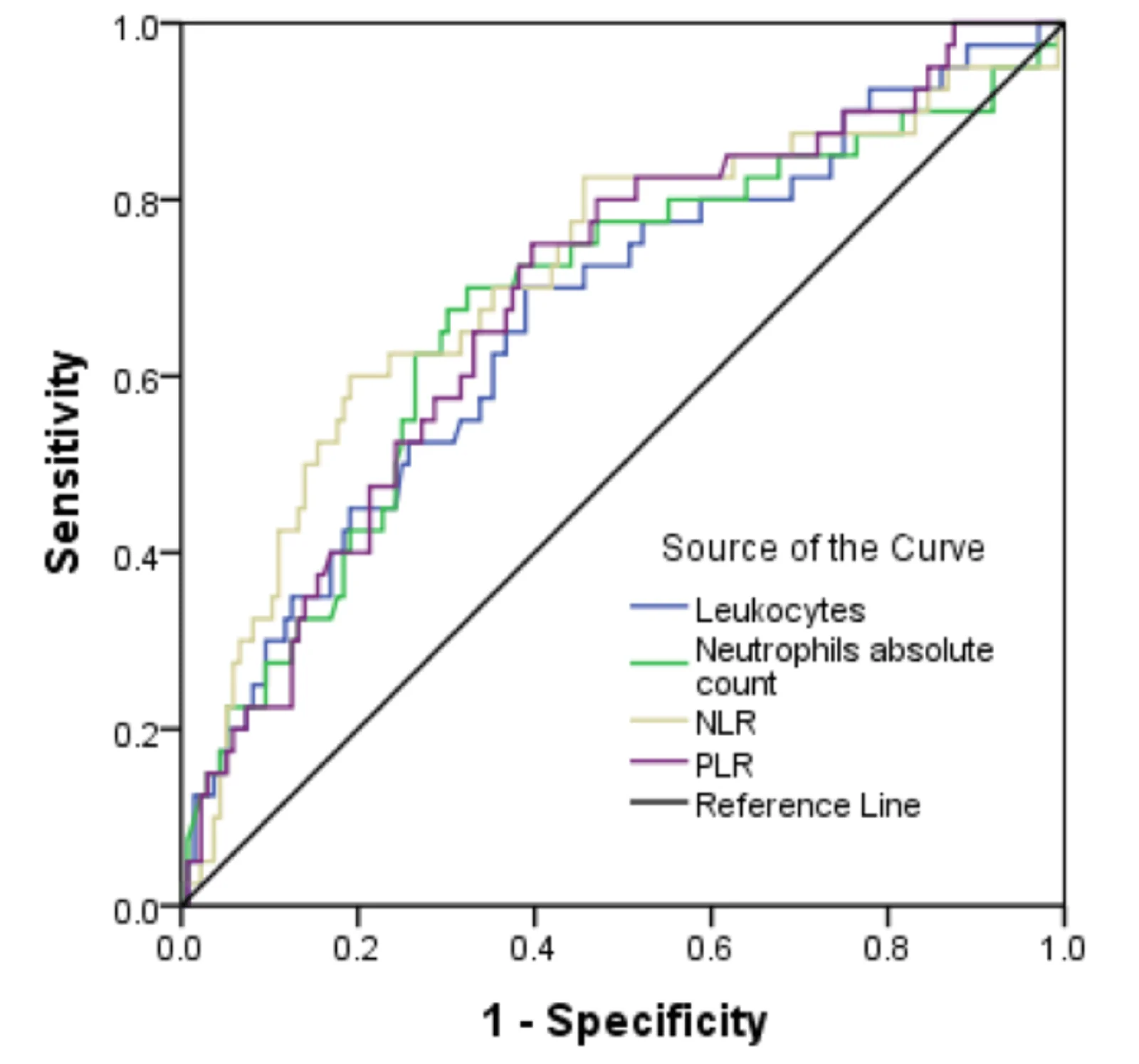
Receiver operating characteristic curve analysis for lab results differentiating complicated vs. non-complicated appendicitis NLR: neutrophil-to-lymphocyte ratio; PLR: platelet-to-lymphocyte ratio

In the present study, receiver operating characteristic (ROC) curve analysis demonstrated that NLR had a better discriminatory ability than PLR for predicting complicated appendicitis in children. Furthermore, NLR gave an area under the curve (AUC) of 0.714 versus 0.690 for PLR, indicating that NLR is a more accurate inflammatory marker for identifying complicated disease. The optimal cut-off value for NLR was 9.85 with 62.5% sensitivity and 76.5% specificity, while PLR had a somewhat lower diagnostic performance with 60% sensitivity and 68.4% specificity. Both biomarkers have a statistically significant predictive value, but the moderate sensitivity of the markers warrants their use as a component of a multivariable model. Rather, these should be combined with clinical assessment, physical examination, and radiological findings to better identify complicated appendicitis preoperatively.

## Discussion

The NLR and PLR have attracted much attention as inexpensive and easy-to-measure indices of inflammation, especially in pediatric appendicitis. These ratios have been specifically studied in children presenting to emergency departments to differentiate complicated from simple appendicitis [3]. Çelik et al. present a retrospective study that used patient data to evaluate the reliability and clinical usefulness of NLR and PLR as early predictors in an attempt to answer the critical need for fast and accurate diagnosis in pediatric emergency settings. The interrelation of the changes in neutrophils and platelets to lymphocytes confirms the scientific basis for using hematological changes in the inflammatory response as markers of severe disease development. The introduction of such biomarkers into clinical practice may help healthcare providers make better decisions that have the potential to shorten the time to diagnosis, make more efficient use of resources, and improve surgical outcomes for pediatric patients. Not only is the NLR a reliable indicator of acute appendicitis in children, but the PLR is also a reliable indicator. Two decades ago, Çelik et al. [3] and Ayeni et al. [4] published their findings. More specifically, we evaluated the performance of these hematological ratios in the detection of acute appendicitis and in the differentiation between simple and complicated forms.

This allowed us to provide valuable insights and bridge the gap between broad diagnostic markers and refined clinical decision-making [4]. Non-invasive prediction of the severity of appendicitis is of immense value in pediatric care, as it can lead to prompt management, optimal resource allocation in emergency settings, and fewer unnecessary surgical investigations. Particularly important is the combination of NLR and PLR as complementary markers, given their sensitivity to systemic inflammatory conditions that tend to differentiate uncomplicated from complicated appendicitis, thus increasing diagnostic accuracy. These associations underscore the significance of these lab-based markers in the everyday diagnostic workup in the end to contribute to more personalized and effective treatment strategies in children presenting with acute abdominal pain.

The severity assessment of pediatric patients with complicated appendicitis can be facilitated by the clinical utility of the readily available biomarkers NLR and PLR, which are strongly associated with the risk of complicated appendicitis. Both NLR and PLR are indicators of underlying inflammatory processes which are more pronounced in complicated cases; thus, the combination of laboratory findings with clinical decision-making is essential in the management of pediatric appendicitis [5]. Other important studies are available showing the importance of NLR and PLR as markers of severity and the possibility to use them to improve diagnostic accuracy [6].

Inclusion of these hematologic indices in the routine diagnostic workup may improve early identification of high-risk patients, allow for timely interventions, and potentially reduce the number of adverse events. Among the NLR diagnostic tools, the most sensitive and specific for the diagnosis of acute appendicitis in children is shown and well presented in the study of Esquivel-Esquivel and Horta-Baas [7].** **This diagnosis is the best way to find out if a child has acute appendicitis or not. This makes it a useful tool that can be added to the clinical evaluation and routine laboratory markers [7]. This is further supported by Begic-Kapetanovic et al. [8], who also confirm NLR's role in initial diagnosis but also its predictive significance in the onset of complications such as perforation or abscess formation.

The authors clearly described that high NLR values have been consistently associated with higher rates of complicated appendicitis, including perforation or abscess formation, and emphasized their prognostic utility in stratifying patients who may require urgent surgical intervention versus those suitable for conservative management. Significantly, the combined use of NLR with other inflammatory indices, e.g., PLR, improves the diagnostic accuracy overall and provides a more detailed assessment of the disease progression, which leads to better tailoring of therapies by the physicians [8]. High NLR values may be beneficial in the differential diagnosis of true acute appendicitis from other causes of abdominal pain, which can help prevent the need for unnecessary surgical interventions in children who do not have appendicitis, as shown in Delgado-Miguel et al. [9].

This tailored strategy not only decreases the physical and psychological stress of unnecessary surgery but also saves costs for healthcare and reduces the risk of postoperative complications. At the same time, a previous finding of Bălănescu et al. showed that higher NLR levels are strongly associated with complicated appendicitis and a higher probability of progression to appendicular peritonitis, emphasizing NLR's usefulness as a prognostic tool in identifying children at risk for severe disease [10].

This study went a step further than the previous studies (Tables 5-6) that investigated the diagnostic role of NLR and directly compared NLR to PLR as a predictive marker for complicated acute appendicitis in children.

**Table 5:** Current and previous studies comparing neutrophil-to-lymphocyte ratio and platelet-to-lymphocyte ratio: complicated vs. non-complicated acute appendicitis

Publication (year)	Setting	Neutrophil-to-lymphocyte ratio			Platelet-to-lymphocyte ratio		
		Cut-off	Specificity (%)	Sensitivity (%)	Cut-off	Specificity (%)	Sensitivity (%)
Current study	Saudi Arabia	9.85	62.5	76.5	216.22	60	68.4
Ayeni et al. (2022) [4]	England	3.8	70	70.3	149.25	61	64
Bălănescu et al. (2022) [10]	Romania	8.39	70.11	73.17	201.4	64.14	63.41
Esquivel-Esquivel and Horta-Baas (2022) [7]	Mexico	5.08	78.9	85.1	-	-	-
Begic-Kapetanovic et al. (2021) [8]	Bosnia	3.48	74	63	-	-	-
Virmani et al. (2018) [11]	India	4.80	60.5	66.7	-	-	-
Çelik et al. (2019) [3]	Turkey	10.4	73	61	284	86	42

**Table 6:** Current and previous studies comparing neutrophil-to-lymphocyte ratio and platelet-to-lymphocyte ratio: complicated and non-complicated acute appendicitis vs. negative appendectomy

Publication (year)	Setting	Neutrophil-to-lymphocyte ratio			Platelet-to-lymphocyte ratio		
		Cut-off	Specificity (%)	Sensitivity (%)	Cut-off	Specificity (%)	Sensitivity (%)
Current study	Saudi Arabia	6.27	54	62.9	165.48	58.5	48.6
Delgado-Miguel et al. (2023) [9]	Spain	2.65	83.8	84.2	-	-	-

Study limitations

It is important to acknowledge the limitations of this study, such as its retrospective design, which depended on pre-existing medical information, and its small sample size. Further prospective, well-designed studies are required in order to evaluate and enhance the accuracy of the related results.

## Conclusions

Our results agree with the previous studies that show the superiority of NLR over PLR in the prediction of complicated acute appendicitis. However, differences in optimal cut-off values and diagnostic accuracy indicate that additional validation is needed, taking into account differences in patient characteristics and study methodology. Use of these biomarkers in diagnostic techniques has the possibility to improve patient outcomes by enabling faster treatment and better utilization of resources. However, further validation and prospective studies are needed to standardize the use of these ratios in pediatric emergency departments.
